# Proteomics-Based Identification and Analysis of Proteins Associated with *Helicobacter pylori* in Gastric Cancer

**DOI:** 10.1371/journal.pone.0146521

**Published:** 2016-01-08

**Authors:** Jianjiang Zhou, Wenling Wang, Yuan Xie, Yan Zhao, Xian Chen, Wenjie Xu, Yan Wang, Zhizhong Guan

**Affiliations:** 1 Molecular Biology Key Laboratory, Guizhou Medical University, Guiyang, Guizhou, China; 2 Department of Clinical Laboratory, Affiliated Hospital of Guizhou Medical University, Guiyang, Guizhou, China; 3 Department of Oncology, Guizhou Cancer Hospital, Guiyang, Guizhou, China; Sapporo Medical University, JAPAN

## Abstract

*Helicobacter pylori* (*H*. *pylori*) is a spiral-shaped Gram-negative bacterium that causes the most common chronic infection in the human stomach. Approximately 1%-3% of infected individuals develop gastric cancer. However, the mechanisms by which *H*. *pylori* induces gastric cancer are not completely understood. The available evidence indicates a strong link between the virulence factor of *H*. *pylori*, cytotoxin-associated gene A (CagA), and gastric cancer. To further characterize *H*. *pylori* virulence, we established three cell lines by infecting the gastric cancer cell lines SGC-7901 and AGS with *cagA*^*+*^ *H*. *pylori* and transfecting SGC-7901 with a vector carrying the full-length *cagA* gene. We detected 135 differently expressed proteins from the three cell lines using proteome technology, and 10 differential proteins common to the three cell lines were selected and identified by LC-MS/MS as well as verified by western blot: β-actin, L-lactate dehydrogenase (LDH), dihydrolipoamide dehydrogenase (DLD), pre-mRNA-processing factor 19 homolog (PRPF19), ATP synthase, calmodulin (CaM), p64 CLCP, Ran-specific GTPase-activating protein (RanGAP), P43 and calreticulin. Detection of the expression of these proteins and genes encoding these proteins in human gastric cancer tissues by real-time PCR (RT-qPCR) and western blot revealed that the expression of *β-ACTIN*, *LDH*, *DLD*, *PRPF19* and *CaM* genes were up-regulated and *RanGAP* was down-regulated in gastric cancer tissues and/or metastatic lymph nodes compared to peri-cancerous tissues. High gene expression was observed for *H*. *pylori* infection in gastric cancer tissues. Furthermore, the *LDH*, *DLD* and *CaM* genes were demethylated at the promoter -2325, -1885 and -276 sites, respectively, and the *RanGAP* gene was highly methylated at the promoter -570 and -170 sites in *H*. *pylori*-infected and *cagA*-overexpressing cells. These results provide new insights into the molecular pathogenesis and treatment targets for gastric cancer with *H*. *pylori* infection.

## Introduction

*Helicobacter pylori* (*H*. *pylori*) causes the most common chronic stomach infection in humans worldwide. Approximately half of the world’s population is infected with *H*. *pylori*, and the majority of colonized individuals develop asymptomatic gastritis. Among infected individuals, approximately 10%-20% of individuals develop peptic ulcer diseases and 1%-3% develop gastric cancer [1,2]. In 1994, *H*. *pylori* was classified as a type I carcinogen for gastric cancer by the World Health Organization’s International Agency for Research on Cancer.

Gastric cancer is one of the most common types of cancers, and more than 70% of new cases and deaths occur in developing countries [3]. Although the global incidence rate has been declining for several decades, gastric cancer remains prevalent in most developing countries, including Japan, Korea and China [4–6]. In 2012, the Chinese Cancer Registry Annual Report indicated that gastric cancer morbidity and mortality are second and third among all malignant tumors, respectively.

The majority of *H*. *pylori* strains carry the *cag* pathogenicity island (*cag*PAI), which contains 27 to 31 genes that encode a bacterial type 4 secretion system (T4SS) [7]. The cytotoxin-associated gene A gene (*cagA*), which is located at the 3′ end of *cag*PAI, encodes the only known bacterial oncoprotein, CagA, which is translocated into host cells by T4SS after bacterial attachment to the stomach. Once inside the host cells, CagA is tyrosine phosphorylated by members of the Abl and Src kinase families and interacts with numerous intracellular effectors, leading to the activation of downstream signaling molecules [8]. In clinical studies, *cagA*-positive strains have been consistently linked to more severe gastric inflammation and ulcers, and a small fraction of individuals develop gastric cancer [9]. However, the mechanisms underlying the association of CagA with cancer have not been elucidated.

Proteomics has emerged as a promising technological platform for the rational identification of biomarkers and novel therapeutic targets for diseases and the determination of the underlying mechanisms of carcinogenesis [10]. However, most of the important proteins detected by proteomics in vitro have not been confirmed in vivo in clinical samples.

DNA methylation and demethylation plays an important role in the development and progression of cancers by blocking the binding of transcription factors to DNA and is occurs nearly exclusively in gene promoter CpG islands [11–13]. Among organs, the stomach exhibits the highest frequency of abnormal CpG island methylation, possibly *H*. *pylori*-mediated [14–16]. Although studies of DNA methylation are increasing, the gene methylation states induced by *H*. *pylori* are not yet clear.

In this work, we aimed to identify specific proteins related to *H*. *pylori* infection using comparative proteomics and characterize the gene expression and CpG island methylation of these proteins in gastric cancer tissues and cells.

## Materials and Methods

### Human tissues

Human tissues were obtained from surgery specimens from 30 gastric cancer patients and matched adjacent cancer tissues and metastatic lymph nodes at Guiyang Medical Hospital, Guiyang China, between January 2009 and June 2010. The diagnoses were confirmed by two pathologists. Among the patients, 23 were male, and 7 were female. The patients ranged in age from 38 to 77 years. Twenty-two patients had intestinal-type adenocarcinoma, and 8 had diffuse-type adenocarcinoma. The study protocol was approved by the Ethics Committee of Guiyang Medical Hospital, and all subjects provided written informed consent.

### Cell culture

The human gastric carcinoma cell line AGS (ATCC CRL-1739TM) and SGC-7901 cells were purchased directly from American Type Culture Collection (ATCC) and Cell Bank of Type Culture Collection of the Chinese Academy of Sciences (Shanghai, China), respectively, and passaged for less than 3 months in our laboratory after receipt. Cells were cultured in Roswell Park Memorial Institute (RPMI) 1640 medium supplemented with 10% heat-inactivated fetal bovine serum, 100 U/ml penicillin and 100 μg/ml streptomycin (Gibco, Grand Island, NY, USA) at 37°C in a humidified incubator with 5% CO_2_.

### *H*. *pylori* culture

*H*. *pylori* strain NCTC11637 (ATCC 43504, a gift from the Chinese Center of *Helicobacter pylori* strain Management and Preservation, Beijing, China), which is *cagA* positive, was grown in selective medium on a Columbia agar plate containing 10% fetal calf serum and *H*. *pylori* Selective Supplement (Oxoid Ltd, England) at 37°C under microaerobic conditions.

### Cell infection with *H*. *pylori*

AGS and SGC-7901 cells (5×10^5^) were seeded in 6-well plates for 24 h and infected with *H*. *pylori* for 6 h and 12 h at a multiplicities of infection (MOIs) of 1:100, 1:500 and 1:1000, respectively. Cells were infected for 12 h at a MOI of 1:1000 with *H*. *pylori* boiled for 15 min as controls.

### Construction of the expression vector pcDNA3.1/*cagA*

DNA was extracted from *H*. *pylori*, and the full-length *cagA* sequence was synthesized by PCR and cloned into pMD18-T plasmids to construct pMD18-T/*cagA*. The *cagA* gene was identified by sequencing (GQ161098). pMD18-T/*cagA* was digested with the restriction enzymes *Pst*I and *Bam*HI, and *cagA* was ligated into pcDNA3.1/Zeo (-) (Invitrogen, USA) to construct the eukaryotic expression vector pcDNA3.1/*cagA*. The sequences of the primers are provided in Table 1.

**Table 1:** Primers used in PCR and quantitative RT- PCR.

Gene	Primer sequence	Length (bp)	GenBank accession no.
***cagA***^a^	Sense: 5′-ACAATGACTAACGAAACCA-3′	3467	GQ161098
	Antisense: 5′-TTTTGGTAT TCCTTAATCCT-3′		
***cagA***^b^	Sense: 5′-AATACACCAACGCCTCCAAG-3′	397	GQ161098
	Antisense: 5′- AAAAGCTTCTGCAGAGCTGGGAGGTGTG-3′		
***β-ACTIN***	Sense: 5′-TGGAGAAAATCTGGCACCAC-3′	190	BC 016045
	Antisense: 5′-GAGGCGTACAGGGATAGCAC-3′		
***LDH***	Sense: 5′-ACGTCAGCAAGAGGGAGAA-3′	194	NM_005566.3
	Antisense: 5′-AACCGCTTCCAATAACACG-3′		
***DLD***	Sense: 5′-TTCCCATTTGCTGCTAACA-3′	214	NM_000108.3
	Antisense: 5′-CTGATAAGGTCGGATGTGC-3′		
***PRPF19***	Sense: 5′-GGACGGAGATTCTTCACTTTACAG-3′	183	NM_014502.4
	Antisense: 5′-CAAACCCTAATTCTACCCCTCTACT-3′		
**ATP synthase**	Sense: 5′-GCTGCCACTCAACAACTT-3′	113	NM_001001937.1
	Antisense: 5′-AGACCCGCATAGATAACAG-3′		
***CaM***	Sense: 5′-TGGAGACGGACAAGTCAACTAT-3′	243	NM_006888.3
	Antisense: 5′-GACAGGACCACCAACCAATAC-3′		
***p64 CLCP***	Sense: 5′-CCTTTGCCACTTGCTCAT-3′	198	NM_001288.4
	Antisense: 5′-CCTTTGCCACTTGCTCAT-3′		
***RanGAP***	Sense: 5′-AGGAGGAAGATGAGGAAGAGG-3′	180	NM_002883.2
	Antisense: 5′-AAGCCAGGAAGGTGGAGAC-3′		
**calreticulin**	Sense: 5′-GCCGAGCCTGCCGTCTACTT-3′	238	NM_004343.3
	Antisense: 5′-TGAACTGCACCACCAGCGTCT-3′		
***HPRT***	Sense: 5′-TGAGGATTTGGAAAGGGTGT-3′	118	NM_000194.2
	Antisense: 5′-GAGCACACAGAGGGCTACAA-3′		

^a^ Primers used to amplify full-length ca*gA*.

^b^ Primers used to detect *cagA* expression in cells. LDH, L-lactate dehydrogenase. *DLD*, Dihydrolipoamide dehydrogenase. *PRPF19*, pre-mRNA processing factor 19 homolog. *CaM*, calmodulin. *p64 CLCP*, nuclear chloride ion channel protein. *RanGAP*, Ran-specific GTPase-activating protein. *HPRT*, Hypoxanthine-guanine phosphoribosyltransferase.

### Cell transfection with pcDNA3.1/*cagA*

SGC-7901 cells were incubated for 12 h in 6-well plates to obtain 80% confluent cells. The cells were then transfected according to the manufacturer’s instructions. After 48 h, the cells were divided 1:10 into new 6-well plates and incubated for an additional 24 h and then maintained in selective zeocin-containing (250 μg/mL) standard medium for 2 weeks until clone formation. Cells transfected with empty vector pcDNA3.1/Zeo (-) were used as controls.

### Protein extraction and western blot

Protein extracts were prepared by resuspending cell pellets in a RIPA buffer or homogenizing 200 mg tissues in a RIPA buffer. A total of 30–50 μg of protein extract was subjected to SDS-PAGE gel electrophoresis, transferred to a polyvinylidene difluoride (PVDF) membrane (Millipore, Billerica, MA, USA) and blotted overnight with mouse monoclonal anti-CagA antibody (1:800, sc-28368), mouse monoclonal anti-phosphotyrosine antibody (PY99, 1:300, sc-7020), rabbit polyclonal anti-beta actin antibody (1:500, ab189073), rabbit polyclonal anti-LDH antibody (1:350, ab125683), mouse monoclonal anti-DLD (1:800, sc-376890), rabbit polyclonal anti-PRPF19 (1:400, ab27692), mouse monoclonal anti-ATP synthase antibody (1:300, ab54880), rabbit polyclonal anti-calmodulin anbody (1:250, ab208911), rabbit polyclonal anti-RanGAP antibody (1:200, ab92360), rabbit polyclonal anti-p64CLCP antibody(1:300, ab28722), rabbit polyclonal anti-calreticulin antibody (1:350, ab4) and mouse monoclonal anti-glyceraldehyde-3-phosphate -dehydrogenase antibody (GAPDH, 1:8000) from Santa Cruz (CA, USA), Abcam (Cambridge, UK) and CangChen (Shanghai, China), respectively, in 5% BSA in Tris-buffered saline and 0.01% Tween-20. Peroxidase-conjugated secondary antibodies (1:5000, SC-2371, Santa Cruz, CA, USA) were used and developed with the chemiluminescence reagent ECL Plus using hyperfilm (Amersham Biosciences, Buckinghamshire, UK). Quantification of the western blots was performed using Quantity One software. Each experiment was performed 3 times, and a representative result is shown.

### Protein extraction and two-dimensional gel electrophoresis

Cell pellets were dissolved in cell lysis buffer overnight at 4°C, and protein was precipitated with three volumes of ice-cold acetone by incubating at 4°C for 2 h. The samples were then centrifuged at 20,000 rpm for 30 min, and the pellets were resuspended in cell lysis buffer and stored at 4°C overnight.

A total of 800 μg of protein was adjusted to a volume of 250 μL with rehydration solution, and isoelectric focusing (IEF) was performed using an Ettan IPGphor II Isoelectric Focusing system (Amersham Biosciences) according to the manufacturer’s instructions. The protocol for IEF was 300 V for1 h, 500 V for 2.5 h, 1,000 V for 2 h; 8,000 V for 8 h, 60 kVh (total).

After completing IEF, the IPG strips (Amersham Biosciences) were equilibrated in equilibration buffer for 15 min and placed on a 12% SDS-PAGE gel for two-dimensional electrophoresis at 30 mA/gel. The resulting SDS-PAGE gel was fixed in 20% TCA for 30 min and then stained with colloidal Coomassie G-250 [5].

The protein spots on the gel were scanned and analyzed automatically. Differentially expressed protein spots were confirmed with Imaging Master 2D 5.0 analytical software (Amersham Biosciences). Student’s t-test was performed for the quantitative analysis of the 2D gels. Differential expression of a specific protein was defined as a ≥2-fold change in spot optical density between the two matched sets in duplicates. The differential spots were then excised from the SDS-PAGE gels for further identification by LC-MS/MS.

### RNA extraction and quantitative RT-PCR

Total RNA was extracted from 50 mg of tissue and treated with DNase I (RNase-free). The genes were amplified with SYBR Green (Applied Biosystems, Australia). Hypoxanthine-guanine phosphoribosyltransferase (*HPRT*) was used as a normalization control, and relative mRNA levels were calculated by a comparative C_t_ method using Step-one software (Applied Biosystems, Australia) [8]. Each sample was assayed in triplicate, and the results are expressed as the mean±SD. The primers used are listed in Table 1.

### DNA extraction and methylation analysis of CpG islands

DNA was isolated from cells and modified with sodium bisulfite using an EZ DNA Methylation-Gold Kit^™^ (Zymo Research, CA, USA) according to the manufacturer’s instructions. Promoter CpG islands were predicted using Methyl Primer Express software and amplified from bisulfite-modified DNA by PCR using the following procedures: denaturing at 96°C for 10 min, followed by 40 cycles of denaturing at 94°C for 40 sec, annealing at 60°C for 40 sec, and elongation at 72°C for 40 sec, with a final extension at 72°C for 10 min. The amplified PCR products were cloned into pMD19-T vectors and sequenced. In addition, DNA isolated from tumor tissues was used to detect *H*. *pylori* 16S *rRNA* gene and *cagA* gene. The primer sequences are listed in Table 2.

**Table 2:** Primers used to amplify promoter CpG islands in methylation-PCR.

Gene	Primer sequence (5′-3′)	Length (bp)
***Hp16S rRNA***	Sense: 5′-GCTAAGAGAT CAGCCTATGTC-3′	118
	Antisense: 5′- CCGTGTCTCAGTTCCAGTGT-3′	
***LDH1***	Sense: 5′-CGGAATAAGGATATGATAGGT-3′	427
	Antisense: 5′-ATCCCTAACTATCTCCTAACTTT-3′	
***LDH2***	Sense: 5′-GGGGTATTTATTAGGTTTGAAGTT-3′	532
	Antisense: 5′-CCTCCTAAAAA TTCACCCATC-3′	
***DLD1***	Sense: 5′-TGTGGATATAGGAGGTGA ATTTT-3′	447
	Antisense: 5′-CAATCAAATCCCAAAAACAATA-3′	
***DLD2***	Sense: 5′-TTTATATGGTTGTTGTAAGGATGAA-3′	473
	Antisense: 5′-CCTTAACCAAAAAACAATACACAC-3′	
***RanGAP1***	Sense: 5′-TTAGTATAGTGGTATGGATGGTAGG-3′	314
	Antisense: 5′-TCTACTAACCCAACCCTACTCTATT-3′	
***RanGAP2***	Sense: 5′-GGGATTGATAGGATATATGGGAT-3′	301
	Antisense: 5′-AATAAATCTAACACCAAAATAACCC-3′	
***CaM1***	Sense: 5′-AAGAGGATTAATTTTTTTTAGGAGG-3′	250
	Antisense: 5′-CAACCTCACCCCACCTAAATA-3′	
***CaM2***	Sense: 5′-GTTGAGGTGGGAGGGTTATTTA-3′	392
	Antisense: 5′-TCCCAACACCACTACCGAA-3′	
***CaM3***	Sense: 5′-TTTTGGTAGTGGTGTTGGGA-3′	285
	Antisense: 5′-AACAAACAAAACAACTAAAAATCTAAA-3′	

*Hp*, *H*. *pylori*; *LDH*, L-lactate dehydrogenase; *DLD*, Dihydrolipoamide dehydrogenase; *RanGAP*, Ran-specific GTPase-activating protein; *CaM*, calmodulin.

### Statistical analysis

Results are expressed as the means ± SD. Statistical analyses were performed using SPSS 15.0 software. One-way analysis of variance (ANOVA) and Student’s t-test were used to analyze the data. *P*<0.05 (two-sided) was considered significant.

## Results

### Introduction of CagA into gastric cancer cells

**Fig 1:**
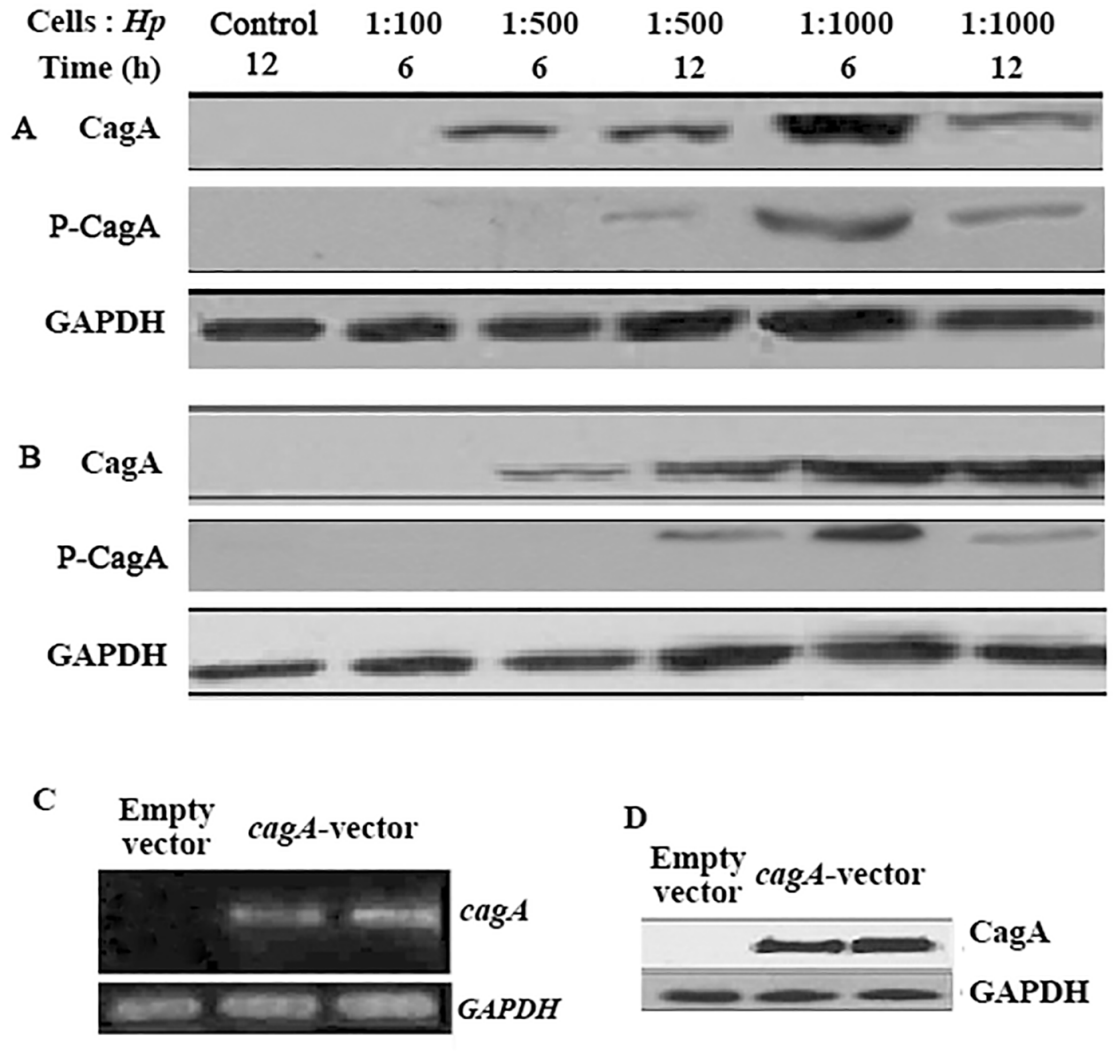
Introduction of CagA into gastric cancer cells. (A and B) Western blot analysis of CagA and phosphorylated CagA in *H*. *pylori*-infected SGC-7901(A) and AGS (B) cells. The cells infected with the indicated ratio of cells to *H*. *pylori* for the indicated time were collected and lysed, and the proteins were separated by SDS-PAGE. Cells infected with *H*. *pylori* boiled for 15 min at a MOI of 1:1000 were used as a control. (C and D) Detection of CagA mRNA and protein in *cagA*-overexpressing SGC-7901 cells by RT-PCR (C) and western blot (D). GAPDH served as the loading control. The data are representative of three independent experiments. *Hp*, *H*. *pylori*; P-CagA, phosphorylated CagA; GAPDH, Glyceraldehyde-3-phosphate- dehydrogenase.

Because CagA of *H*. *pylori* is a critical virulence factor in the development and progression of gastric cancer, CagA was detected in gastric cancer cell lines by RT-PCR and western blot after infection of SGC-7901 and AGS cells with *H*. *pylori* and transfection of SGC-7901 cells with *cagA*-vector. The CagA protein began to appear at a ratio of cells to bacteria of 1:500 in cultured cells at 6 h, and the content was highest at a ratio of 1:1000 at 6 h (Fig 1A and 1B). However, phosphorylated CagA was observed in cells at a ratio of 1:500 after culturing for 12 h, and the highest content was observed in cells at a ratio of 1:1000 after 6 h of culture. CagA mRNA and protein were also observed in stably *cagA*-overexpressing SGC-7901 cells (Fig 1C and 1D). These data suggest that CagA was successfully introduced into the three cell lines and phosphorylated.

### Identification of differential proteins in gastric cancer cells

**Fig 2:**
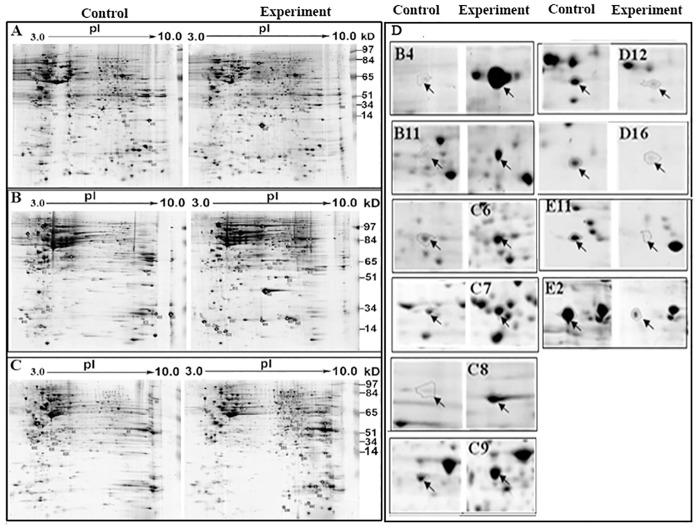
Representative 2-DE maps and magnified image of differential spots in three cell lines. SGC-7901 and AGS cells infected with *H*. *pylori* for 6 h at a MOI of 1:1000 (cell to *H*. *pylori*) and SGC-7901 cells transfected with pcDNA3.1/*cagA* for 48 h were collected and lysed, and the protein concentrations were determined using Bradford colorimetry. A total of 800 μg of protein was loaded for two-dimensional electrophoresis. Cells infected with boiled *H*. *pylori* or transfected with empty vector served as controls for the infected or transfected cells, respectively. (A) SGC-7901 cells infected with *H*. *pylori*. (B) AGS cells infected with *H*. *pylori*. (C) SGC-7901 cells transfected with the *cagA*-vector. (D) Magnified image of 10 differential spots. B4, B11, C6, C7, C8 and C9 spots were up-regulated, whereas D12, D16, E2 and E11 spots were down-regulated. These spots are identified in Table 3.

**Fig 3:**
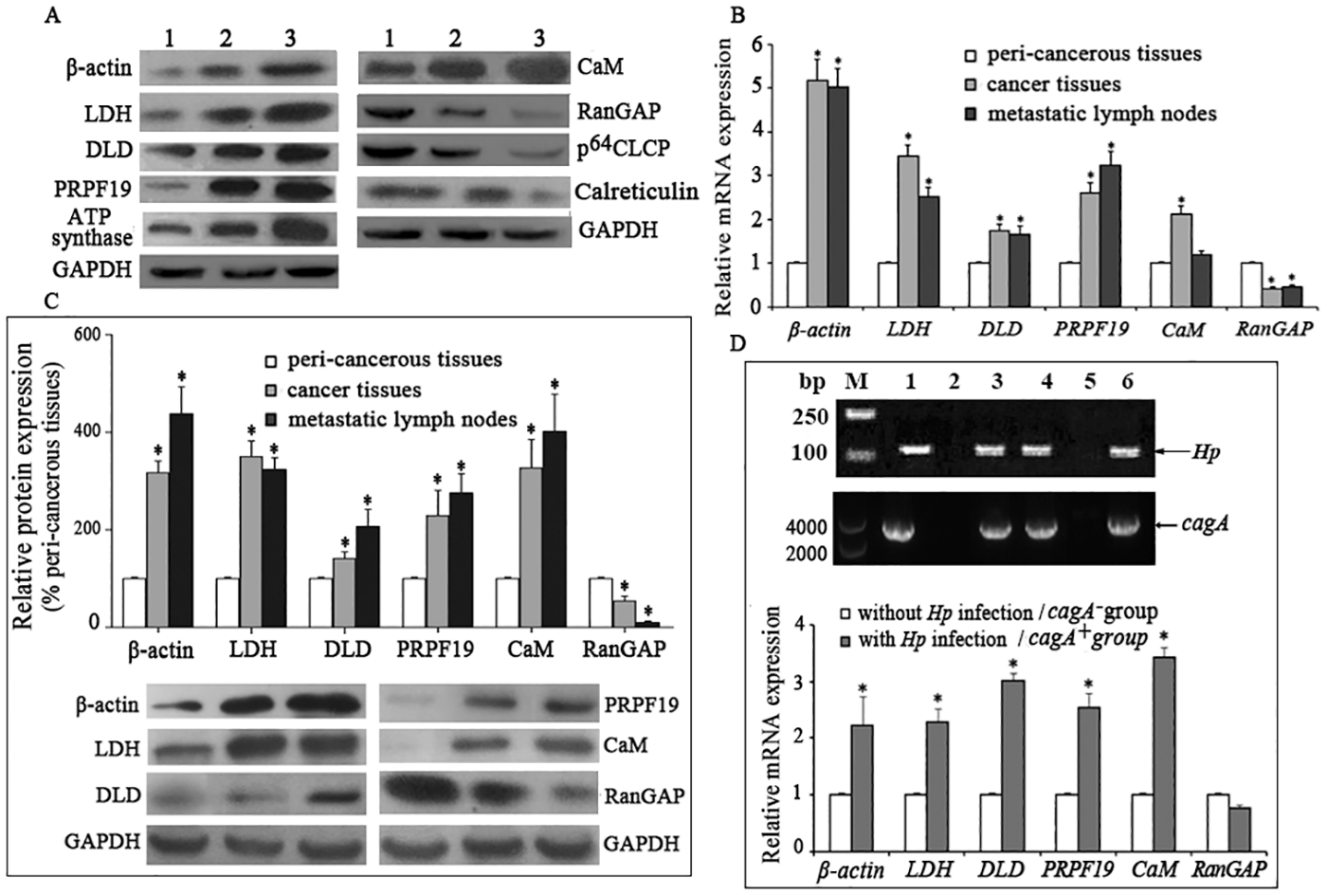
*H*. *pylori* infection promotes the genes and proteins expression in gastric cancer tissues. (A) Western blot analysis of the indicated proteins in the control cells (1), *H*.*pylori*-infected SGC-7901 cells (2) and *cagA*-overexpressed SGC-7901 cells (3). GAPDH served as the loading control. The data are representative of three independent experiments. (B) Quantitative RT-PCR analysis of the indicated genes in 30 gastric cancer tissues. Values are represented as average Ct fold compared to peri-cancerous tissues, where peri-cancerous tissues were set to 1. (C) Western blot analysis of the indicated proteins in 30 gastric cancer tissues. 200 mg tissues were homogenized and total proteins were collected. A total of 50 μg protein extracts were subjected to SDS-PAGE gel electrophoresis. GAPDH served as the loading control. (D) Detection of the *H*. *pylori* 16S *rRNA* gene and *cagA* gene in gastric cancer tissues by PCR (Upper). M represents the DNA molecular weight marker. Lane 1 is the positive control. Lane 2 is the negative control. Lanes 3, 4 and 6 are positive samples. Lanes 5 are negative samples. Quantitative RT-PCR analysis of the indicated genes in gastric cancer tissues with and without *H*. *pylori* infection (Lower). Values are presented as the average Ct fold compared to the group without *H*. *pylori* infection, which was set to 1. The figure presents the average of 30 samples. Data are presented as the means ± SD. Error bars represent standard deviations. LDH, L-lactate dehydrogenase. DLD, Dihydrolipoamide dehydrogenase. PRPF19, pre-mRNA processing factor 19 homolog. RanGAP, Ran-specific GTPase-activating protein. CaM, calmodulin. p64 CLCP, nuclear chloride ion channel protein. *, *P*<0.05 compared to peri-cancerous tissue (B and C) and tissues without *H*. *pylori* infection (D).

After cells were infected with *H*.*pylori* for 6 h at a MOI of 1:1000 or transfected with *cagA*-vector, a proteomic technique was used to create six two-dimensional electrophoresis (2-DE) maps from the three cell lines and their respective controls (Fig 2), and 135 differential spots were detected, of which 73 were up-regulated and 62 were down-regulated. Ten differential spots common to all three cell lines were identified by LC- MS/MS and verified by western blot, including 6 up-regulated proteins: β-actin, LDH, DLD, PRPF19, ATP synthase and calmodulin (CaM), and 4 down-regulated proteins such as RanGAP, p64 CLCP, P43 and calreticulin (Table 3 and Fig 3A).

**Table 3:** Identification of differential proteins in three cell lines by LC-MS/MS.

SpotNo.	Accession No.	Protein	*M*(dalton)	p*I*	Sequencecoverage (%)	Score	Fold change	Function
**B4**	gi|4501885	beta actin	41710.7	5.29	8	30	↑106.355	Skeleton rearrangement
**B11**	gi|13786847	L-Lactate Dehydrogenase	36485.1	5.72	20	90	↑3.3682	Energy metabolism
**C6**	gi|91199540	dihydrolipoamide dehydrogenase	54144	7.95	9	60	↑2.59701	Energy metabolism
**C7**	gi|7657381	pre-mRNA processing factor 19 homolog	55147.4	6.14	15	70	↑2.73432	pre-mRNA splicing
**C8**	gi|4757810	ATP synthase	59714.6	9.16	24	218	↑5.45285	Energy metabolism
**C9**	gi|825635	Calmodulin	17153	4.06	21	40	↑3.55856	Signal transduction
**D12**	gi|895845	p64 CLCP	23592.1	5.12	65	328	↓6.11339	Ion transport
**D16**	gi|542991	Ran-specific GTPase-activating protein	23439.7	5.21	12	30	↓4.55474	Signal transduction
**E11**	gi|833999	P43	49503.1	7.69	31	200	↓7.66244	anti-angiogenesis
**E2**	gi|4757900	calreticulin	48112.8	4.29	55	898	↓11.9006	calcium homeostasis

### *H*. *pylori* infection promotes the expression of differential proteins in gastric cancer tissues

Because *H*. *pylori* selectively colonizes the human stomach to activate a set of pathological processes, the gene expression of 10 differential proteins in 30 human gastric cancer samples was evaluated by quantitative RT-PCR. Of the 10 genes, the expression of *β-ACTIN*, *LDH*, *DLD*, *PRP19* and *CaM* in gastric cancers and/or metastatic lymph nodes were up-regulated compared to peri-cancerous tissues, whereas the expression of *RanGAP* was down-regulated. Similarily, the 6 proteins were also abnormally expressed in the same tissues (Fig 3B and 3C). No significant differences in the expression of the other genes were observed.

*H*. *pylori* colonization in gastric cancer tissues was measured by detecting the 16S *rRNA* gene and *cagA* gene of *H*. *pylori* by PCR. *H*. *pylori* was present in 20 of the 30 samples (positive rate 67%), and all of *H*. *pylori* strains are *cagA* positive. The mRNA levels of *β-ACTIN*, *LDH*, *DLD*, *PRP19 and CaM* were higher in *H*. *pylori*-positive tissues than in the negative samples (Fig 3D).

### *H*. *pylori* induces aberrant DNA methylation in gastric cancer cells

**Fig 4:**
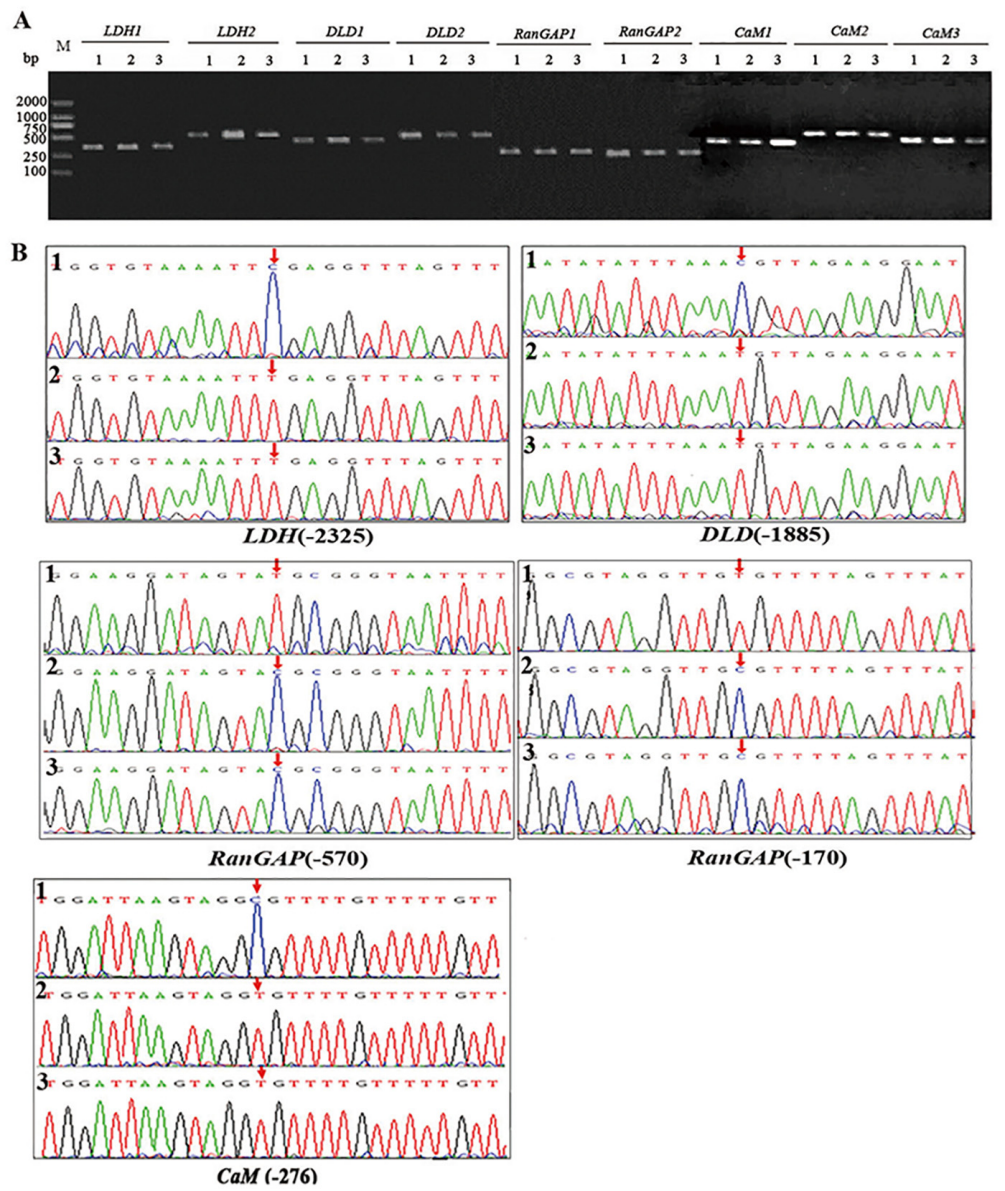
*H*. *pylori* induces aberrant DNA methylation in cells. (A) Electrophoretic analysis of the promoter CpG islands of the indicated genes by bisulfite modification-PCR. (B) Methylation sites of the CpG islands at the indicated gene promoters. *LDH*, L-lactate dehydrogenase. *DLD*, Dihydrolipoamide dehydrogenase. *RanGAP*, Ran-specific GTPase-activating protein. *CaM*, Calmodulin. M, Molecular weight marker. 1, untreated SGC-7901 cells; 2, *H*. *pylori*-infected SGC-7901 cells; 3, *cagA*-overexpressing SGC-7901 cells. The arrows indicate abnormal methylation sites.

In three cell lines, PCR products of the CpG islands of the above genes were successfully acquired following bisulfite treatment and sequenced. In these genes, *LDH*, *DLD* and *CaM* genes were demethylated at the promoter -2325, -1885 and -276 sites, respectively, and the *RanGAP* gene was highly methylated at the promoter -570 and -170 sites (Fig 4).

## Discussion

Accumulative evidence indicates that *H*. *pylori* infection is an important factor for *H*. *pylori*–associated gastric diseases and that CagA is a promoting factor for gastric cancer [17,18]. We constructed three experimental cell lines, including two gastric cancer cell lines infected with *H*. *pylori* (*cagA*^+^) and a gastric cancer cell line overexpressing *cagA*, and determined that CagA began to appear in cells 6 h after infection and for up to 12 h. Subsequently, phosphorylated CagA began to appear, consistent with the injection and subsequent phosphorylation of CagA into gastric epithelial cells by the T4SS of *H*. *pylori* after infection of the human gastric mucosa. Phosphorylated CagA activates downstream signaling pathways and plays a pathological role. We also observed CagA in cells stably transfected with the *cagA*-vector. These data confirm the successful construction of the three experimental cell lines.

Proteomics has been used to study the relationship between *H*. *pylori* infection and gastric diseases, and many differentially expressed proteins have been identified [19–22]. However, the association of these proteins with CagA and their expression in human gastric cancer tissues remain unclear. We obtained a total of 135 differential spots from the three cell lines, of which 73 were up-regulated and 62 were down-regulated. Ten differential spots were common to all three cell lines, including 6 up-regulated proteins (β-actin, LDH, DLD, PRP19, ATP synthase, and CaM) and 4 down-regulated proteins (p64 CLCP, RanGAP, P43 and calreticulin), and the 10 proteins’ expression were verified by western blot in these cell lines. These proteins are involved in energy metabolism, skeleton rearrangement, pre-mRNA processing, signal transduction, and other proteins closely associated with the development and progression of many human cancers [23,24]. We quantitatively detected the expression of the genes encoding these 10 proteins in human gastric cancer tissues, which revealed that *β-ACTIN*, *LDH*, *DLD*, *PRP19* and *CaM* were consistently highly expressed and *RanGAP* was poorly expressed in both cancer tissues and/or metastatic lymph nodes compared to peri-cancerous tissue. The aberrant expression of these proteins was also verified by western blot in gastric cancer and metastatic lymph nodes. Next, we found that *cagA*^+^ *H*. *pylori* colonized in 20 of 30 gastric cancer tissues and promoted or inhibited the expression of these genes in vivo.

LDH and DLD are closely correlated with energy metabolism. Disorder of energy metabolism is considered an important factor for the development and progression of cancer. In contrast to normal cells, cancer cells exhibit increased dependence on the glycolytic pathway with sufficient oxygen, termed aerobic glycolysis. A large quantity of pyruvic acid, a final product of the pathway, is converted to lactic acid by LDH, promoting the glycolytic pathway and energy metabolism imbalance [25]. Lactic acid can also decrease the pH of the cell microenvironment, thus increasing neovascular response to angiogenesis factors to promote tumor cell metastasis [26,27]. Consistent with our results, *LDH* was highly expressed in cancer tissues in 61.8% of patients with gastric cancer; patients with LDH overexpression had shorter survival compared to patients with low expression [28]. Kim *et al*. [29] observed that *DLD* expression increases with tumor progression, thus providing more energy for tumor cell growth. Therefore, high expression of LDH and DLD is a crucial factor for the development and progression of gastric cancer, and *H*. *pylori* infection promotes the expression of these two genes in gastric cancer tissues.

Beta-actin, a key component of the cytoskeleton, maintains the structure, motion and division of cells under normal condition. *β-ACTIN* is abnormally expressed in many diseases [30]. The PRPF19 protein is believed to function in pre-mRNA splicing. Ubiquitination of PRPF19 could lead to DNA damage, and abnormal DNA repair is an important cause of tumorigenesis [31]. Calmodulin is a calcium-binding protein that regulates many signaling pathways and thus participates in cell proliferation, mitosis and gene transcription. Calmodulin promotes cell proliferation in liver carcinomas in combination with PI3K and the transition from G1 to S phase in combination with cyclin E [32]. An inhibitor of calmodulin arrests cells in G1 phase to inhibit cell division [33]. Consistent with these results, we determined that *H*. *pylori* may participate in cancer development of gastric tissues by inducing the high expression of these three proteins.

In addition, we observed down-regulation of the *RanGAP* gene in cancer tissues with *H*. *pylori* infection. RanGAP is a GTPase-activating protein. After hydrolysis of GTP to GDP by a GTPase, active RanGTP becomes inactive RanGDP, leading to the blockade of cell signaling to inhibit cancer progression [34]. Similarly, decreased RanGAP activity induced by ubiquitination promotes cancer development [35].

DNA methylation can induce abnormal gene expression. *H*. *pylori* infection increases the methylation levels of some genes and results in carcinogenesis of the gastric mucosa [36,37]. DNA methylation usually appears in the CpG islands of gene promoters. We detected the methylation status of the CpG islands of these genes in the constructed cell lines and determined that the *LDH*, *DLD* and *CaM* genes were demethylated at the promoter -2325, -1885 and -276 sites, respectively, and the *RanGAP* gene was highly methylated at the promoter -570 and -170 sites, consistent with the high expression of the *LDH*, *DLD* and *CaM* genes and the low expression of the *RanGAP* gene in gastric cancer tissues with *cagA*^+^ *H*. *pylori* infection.

In conclusion, these results suggest that *H*. *pylori* infection, via CagA translocation, may induce the aberrant methylation of these genes to lead to dysfunctional gene expression in gastric cancer tissues and cells. Our results provide a new understanding of the molecular pathogenesis of gastric cancer with *H*. *pylori* infection. Future studies will explore the effects of these altered methylation patterns on the pathogenesis of gastric cancer in clinical samples.
